# Topoisomerase IIIβ Deficiency Induces Neuro-Behavioral Changes and Brain Connectivity Alterations in Mice

**DOI:** 10.3390/ijms222312806

**Published:** 2021-11-26

**Authors:** Faiz Ur Rahman, You-Rim Kim, Eun-Kyeung Kim, Hae-rim Kim, Sang-Mi Cho, Chin-Soo Lee, Su Jin Kim, Kimi Araki, Ken-ichi Yamamura, Mi-Ni Lee, Seul Gi Park, Won-Kee Yoon, Kihoon Lee, Young-Suk Won, Hyoung-Chin Kim, Younghee Lee, Ho-Young Lee, Ki-Hoan Nam

**Affiliations:** 1 Laboratory Animal Resource Center, Korea Research Institute of Bioscience and Biotechnology, Yeongudanji-ro 30, Cheongju 28116, Korea; khanfaiz@kribb.re.kr (F.U.R.); kyr0323@gmail.com (Y.-R.K.); k9845029@kribb.re.kr (E.-K.K.); kz613@kribb.re.kr (H.-r.K.); sangmi@kribb.re.kr (S.-M.C.); chinsoo@kribb.re.kr (C.-S.L.); minilee@kribb.re.kr (M.-N.L.); seulgi@kribb.re.kr (S.G.P.); wkyoon@kribb.re.kr (W.-K.Y.); kihoon@kribb.re.kr (K.L.); yswon@kribb.re.kr (Y.-S.W.); hckim@kribb.re.kr (H.-C.K.); 2 Department of Biochemistry, College of Natural Sciences, Chungbuk National University, Chungdae-ro 1, Cheongju 28644, Korea; 3 Department of Nuclear Medicine, Seoul National University Bundang Hospital, Gumi-ro 166, Seongnam 463-707, Korea; tokimsujin@gmail.com; 4 Institute of Resource Development and Analysis, Kumamoto University, Honjo 2-2-1, Kumamoto 860-0811, Japan; arakimi@gpo.kumamoto-u.ac.jp (K.A.); yamamura@gpo.kumamoto-u.ac.jp (K.-i.Y.)

**Keywords:** Top3β, brain connectivity, behavioral impairments, anxiety, depression, circadian activity, neuropsychiatric disorders

## Abstract

Topoisomerase IIIβ (Top3β), the only dual-activity topoisomerase in mammals that can change topology of both DNA and RNA, is known to be associated with neurodevelopment and mental dysfunction in humans. However, there is no report showing clear associations of Top3β with neuropsychiatric phenotypes in mice. Here, we investigated the effect of Top3β on neuro-behavior using newly generated Top3β deficient (*Top3β^−/−^*) mice. We found that *Top3β^−/−^* mice showed decreased anxiety and depression-like behaviors. The lack of Top3β was also associated with changes in circadian rhythm. In addition, a clear expression of Top3β was demonstrated in the central nervous system of mice. Positron emission tomography/computed tomography (PET/CT) analysis revealed significantly altered connectivity between many brain regions in *Top3β^−/−^* mice, including the connectivity between the olfactory bulb and the cerebellum, the connectivity between the amygdala and the olfactory bulb, and the connectivity between the globus pallidus and the optic nerve. These connectivity alterations in brain regions are known to be linked to neurodevelopmental as well as psychiatric and behavioral disorders in humans. Therefore, we conclude that Top3β is essential for normal brain function and behavior in mice and that Top3β could be an interesting target to study neuropsychiatric disorders in humans.

## 1. Introduction

DNA topoisomerases are “magicians of the DNA world”. They are ubiquitous enzymes that can control topological problems associated with DNA replication, transcription, recombination, and chromatin remodeling by incorporating transient single- or double-strand breaks into DNA [1,2,3]. Seven distinct topoisomerases enzymes (TOP1, TOP1mt, TOP2α, TOP2β, TOP3α, TOP3β, and Spo11) acting on a broad range of deoxyribonucleic acid and ribonucleic acid substrates have been identified so far in mammalian cells [3,4]. Of all members of the DNA topoisomerase family, topoisomerase 3β (Top3β) is the newest member identified both in humans and mice [5,6]. This type 1A topoisomerase has been found to have dual activities, capable of changing the topology of both DNA and RNA in animals [7]. Emerging evidence has shown that mutations in the *TOP3β* gene contribute to neurodevelopmental disorders associated with schizophrenia and cognitive defects in humans [8]. Moreover, in schizophrenia and autism patients, de novo single nucleotide variants of the TOP3β gene have been observed, highlighting its vital roles in genetic and neural complexity [9,10]. Tarsitano et al. [11] have reported that patients with duplicated TOP3β-containing regions exhibit symptoms such as mild intellectual disability/developmental delay, microcephaly, and variable phenotype. TOP3β mutations including smaller genomic deletions are also linked to schizophrenia, autism [12], epilepsy [13], cognitive impairment, and facial dimorphism in human [14]. In distal deletion syndrome characterized by mental retardation and cognitive dysfunction, TOP3β is frequently deleted in the 22q11.2 [15].

Beyond these roles, Top3β has an essential role in the lifespan of mice. Although Top3β deficient mice are viable with normal development to maturity, they display reduced mean lifespan relative to their *Top3β^+/+^* littermates [16]. Moreover, mice lacking Top3β exhibit chromosomal abnormalities and increased apoptosis that might lead to progression of autoimmunity [17]. A recent study published by Joo et al. [18] stated that top3β is essential for normal brain function and that its defect can cause cognitive impairment in mice. We hypothesize that Top3β could affect behavioral phenotype and neurological abnormalities in mice.

To test this hypothesis and to better understand the roles of Top3β in mental disorders, we analyzed neuro-behavioral phenotypes of our newly generated Top3β deficient (*Top3β^−/−^*) mice. We report that Top3β deficiency is associated with alterations of neuro-behavior activity, circadian rhythm, and brain connectivities in mice. These results suggest that Top3β is an interesting target to better understand human mental disorders including schizophrenia and autism.

## 2. Results

### 2.1. Generation of Top3β Mutant Mice

**Figure 1:**
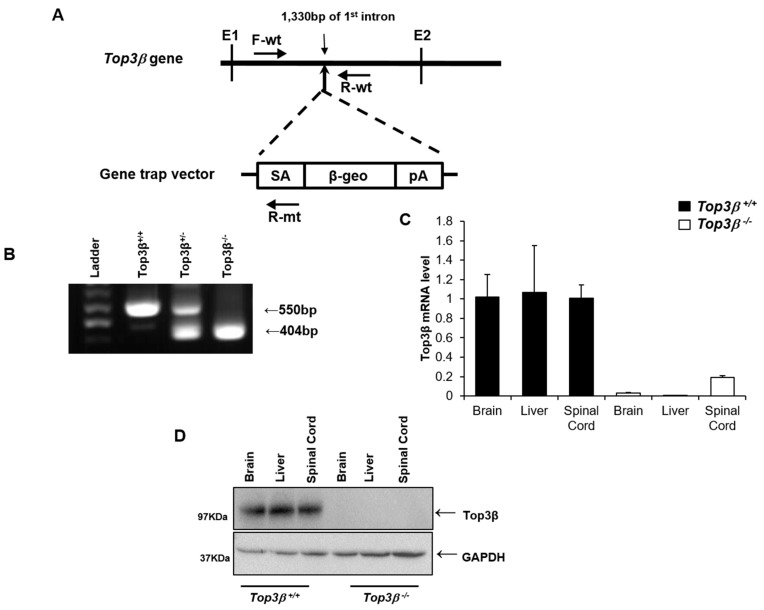
Generation of *Top3β* gene knock-out mouse using a gene trapping method. (**A**) Schematic structure of the Top3β genome locus and the vector insertion site. Locations used for genotyping primers are shown with black arrows. Exons 1 and 2 are shown as E1 and E2, respectively. (**B**) Genotyping results of *Top3β^−/−^* mice. The mutant allele has a PCR product of 404 base pairs using primer pair of F-wt and R-mt and the wild-type allele has a PCR product of 505 base pairs using primer pair of F-wt and R-wt. (**C**) Examination of samples of brain, liver, and spinal cord RNAs with qRT-PCR analysis. (**D**) Western blot analysis of Top3β protein in brain, liver, and spinal cord lysates from *Top3β^+/+^* and *Top3β^−/−^* mice. GAPDH was used as an internal control (lower panel). Full gel images are presented in Supplementary Materials (Figure S1). SA, splice acceptor; PA, polyadenylation.

The insertion site of the gene trap vector (pU-21T) on the genome in the embryonic stem (ES) cell line KTPU8 was confirmed to be between 1330 and 1331 base pairs of intron 1 (Figure 1A). Three specific PCR primers were designed for genotyping. Their approximate positions are indicated in Figure 1A. Primers used for genotyping analysis in this study are listed in the Section 4. Chimeric mouse was produced by co-culturing mutant ES cells with a morula from ICR mice. The chimeric mouse was mated with a C57BL/6J wild type mouse to obtain founder mutant mouse. Representative PCR genotyping results for Top3β mutant mice are shown in Figure 1B. RT-PCR analysis showed that Top3β mRNA was undetectable in brains, livers, and spinal cords from *Top3β^−/−^* mice. However, it was readily detectable in the tissues of their wild (*Top3β^+/+^*) littermates (Figure 1C). These results confirmed the absence of complete Top3β message. Western blot analysis (for full blot, see Supplementary Figure S1) also confirmed the absence of Top3β protein expression in brains, livers, and spinal cords of *Top3β^−/−^* mice, while Top3β protein expression was detected in the organs of wild (*Top3β^+/+^*) littermates (Figure 1D).

### 2.2. Deficiency of Top3β Does Not Affect Mouse Body Weight Gain

In contrast to mice deficient in DNA topoisomerase IIIα that exhibit embryonic lethality [19], mice lacking Top3β are viable and fully mature with shorter life spans [16]. However, *Top3β^−/−^* mice generated by gene trap in this study showed normal life spans (data not shown). When we measured body weights of these mice from 4 to 16 weeks of age, there was no significant difference in body weight gain between *Top3β^−/−^* and *Top3β^+/+^* mice (male or female) (Supplementary Figure S2).

### 2.3. Top3β^−/−^ Mice Show Less Anxiety-like Behavior

**Figure 2:**
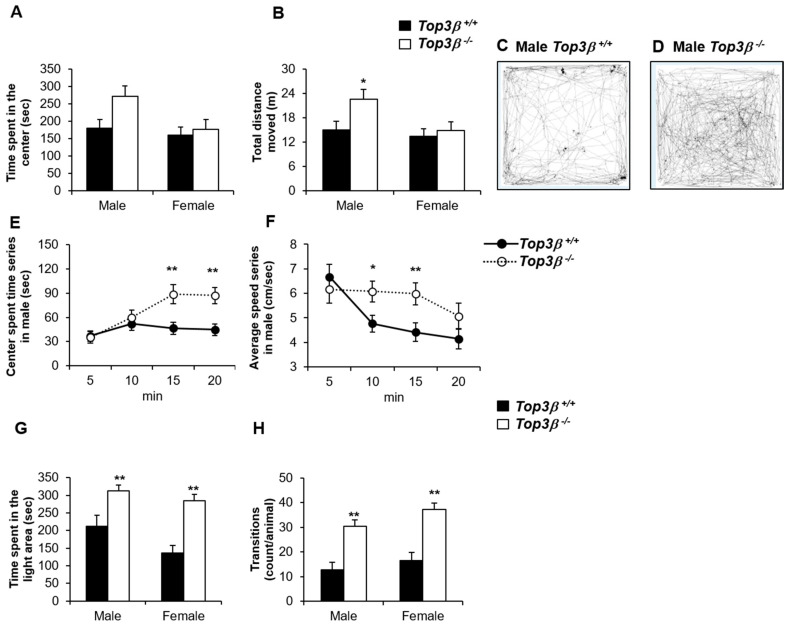
Anxiety-like behavior is lower in *Top3β^−/−^* mice. Open field experiments were carried out with 9 week old *Top3β^−/−^* and Top3β^+/+^ mice (**A**–**F**). (**A**) Time spent at the center area, (**B**) Total distance moved on the arena. Both A and B were significantly decreased in male *Top3β^−/−^* mice than in male Top3β^+/+^ mice. (**C**) A representative image showing the trace of movement for 20 min for male Top3β^+/+^ in the open field test. (**D**) A representative image showing the trace of movement for 20 min of male *Top3β^−/−^* mice in the open field test. (**E**,**F**) Graphs showing the time spent in the center of the arena (**E**) and the average speed in the arena (**F**) every 5 min with a total of 20 min of observation. *Top3β^−/−^* mice showed a gradual increase of center spent time without showing decreased average speed as time elapsed. Light-dark box experiment was performed with 20 weeks old *Top3β^−/−^* and Top3β^+/+^ mice (**G**,**H**). (**G**) Both sexes of *Top3β^−/−^* mice showed significantly longer time spent in the light area and higher number of movement transitions between boxes than those of Top3β^+/+^ mice (**H**). For the open field test, six mice were used for each group (female or male wild or mutant mice). For the light-dark box test, eight mice were used for each group except for the male mutant group which used seven mice for the test. * *p* < 0.05; ** *p* < 0.01.

To see any phenotypic changes in anxiety-like behavior of *Top3β^−/−^* mice, an open field test was performed at the age of 9 weeks. Although there was no significant difference compared with male control mice, male *Top3β^−/−^* mice spent a slightly longer time in the center zone while male mutant mice moved with faster speed on the arena, resulting in longer distance moved than wild-type (WT) littermates (Figure 2A–D). However, there were no significant differences in female mutant mice for all of these parameters. In addition, when results were analyzed for each 5-min segment in a total observation period of 20 min, the time spent at the center area of the arena increased with time for male *Top3β^−/−^* mice, whereas WT male mice showed stable time spent at the center area during the whole observation time (Figure 2E). The average moving speed on the arena for male *Top3β^−/−^* mice was not reduced until the 4th quarter, whereas the speed was reduced after the 1st quarter of the observation period for WT male mice (Figure 2F). On the other hand, there was no significant difference in any parameter between mutant *Top3β^−/−^* and WT *Top3β^+/+^* female mice in the open field test. These data indicate that male, but not female *Top3β^−/−^* mice have less anxiety-like behavior and higher locomotive activity.

### 2.4. Top3β^−/−^ Mice Exhibit Hyperactivity Based on the Light-Dark Box Test

To further compare and explore anxiety-related response, light-dark box experiments with dark start were conducted with mutant *Top3β^−/−^* and WT *Top3β^+/+^* mice. Both male and female *Top3β^−/−^* mice showed significantly shorter latencies to the first exit from the dark box, longer time spent in the light box, and a higher number of transitions between dark and light boxes (Figure 2G,H) compared to WT mice. These data indicate that both male and female *Top3β^−/−^* mice have significantly decreased anxiety-like behaviors and increased locomotor activities compared to their WT littermates.

### 2.5. Top3β^−/−^ Mice Show Decreased Depression-like Behavior

**Figure 3:**
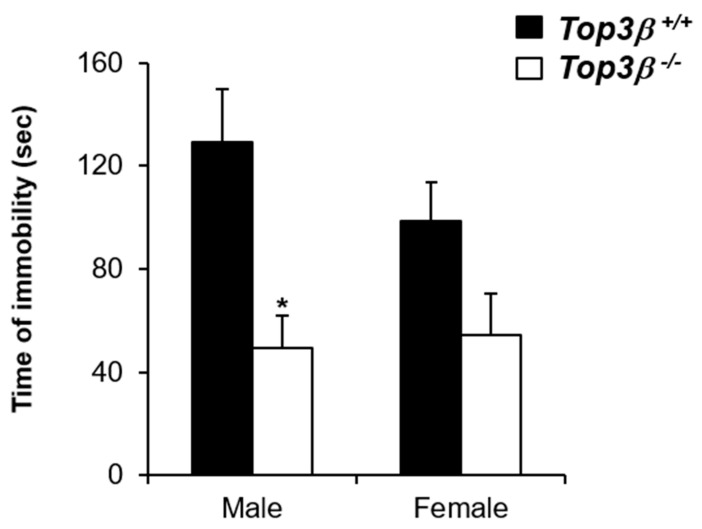
*Top3β^−/−^* mice show decreased depression-like behavior in the forced swim test. The forced swim test was performed with 20-week-old mice. Both male and female *Top3β^−/−^* mice showed reduced immobility time in the forced swim test. Six mice were used for each group except for the male mutant group which used five mice for the test. * *p* < 0.05.

Furthermore, we performed the forced swim test to investigate whether *Top3β^−/−^* mice had any depression-like behavior. Behavioral depression tests such as the forced swim test (FST) are frequently used to assess and quantify the extent to which a mouse model exhibits a depression-like phenotype [20]. *Top3β^−/−^* and Top3β^+/+^ mice at 20 weeks old were subjected to the forced swim test and the time of immobility in water was measured. Male *Top3β^−/−^* mice swam for longer time and hardly gave up swimming compared to their WT counterparts. However, in female mutant mice, no significant differences were observed when compared with their WT controls. With significantly shorter immobility time in the forced swim test, male *Top3β^−/−^* mice showed a reduction in despair state (Figure 3), indicating that Top3β deficiency could induce decreased depression-like phenotype in male mice.

### 2.6. Top3β Deficiency Induces Alteration in Circadian Activity

**Figure 4:**
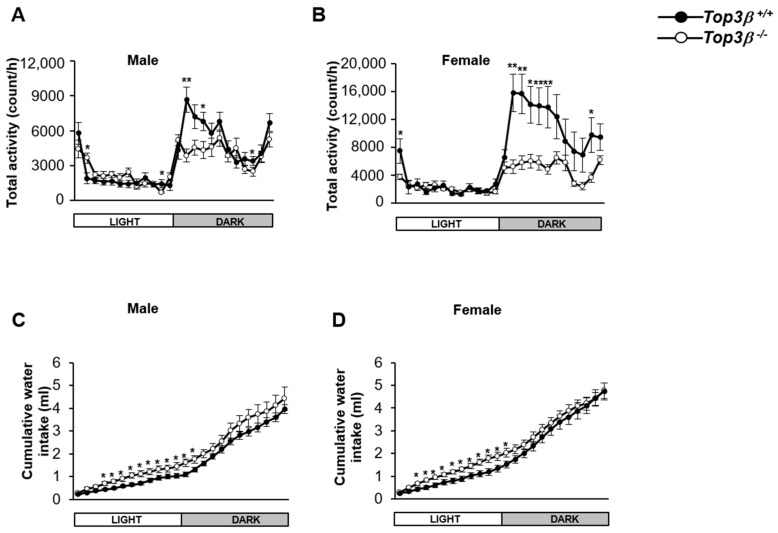
*Top3β^−/−^* mice show altered circadian activity compared to *Top3β^+/+^* mice. Activities of *Top3β^−/−^* mice at 11 weeks of age were analyzed for 24 h with indirect calorimetry using an activity monitoring system. Total movement (**A**,**B**) and water intake (**C**,**D**) for each hour during light and dark times were analyzed for male (**A**,**C**) and female (**B**,**D**) mice. *n* = 6 for each group. * *p* < 0.05; ** *p* < 0.01.

There is a clear association between depression and altered circadian activities [21,22]. Thus, we investigated the effect of Top3β on circadian behavior in *Top3β^−/−^* mice. In the observation using an indirect calorimeter equipped with sensors for activity measurement, both male and female *Top3β^−/−^* mice showed decreased total movement during a dark time compared to WT mice (Figure 4A,B) although there was no change in circadian rhythm, the sleep/wake cycle. Both male and female *Top3β^−/−^* mice also showed increased water intake during a light time compared to WT mice (Figure 4C,D). Taken together, these results suggest that Top3β deficiency not only affects anxiety/depression-related behaviors, but also disrupts circadian activities during light and dark time periods of a day in mice.

### 2.7. Top3β^−/−^ Mice Exhibit Neurological Defects and Altered Neuromuscular Functions

**Figure 5:**
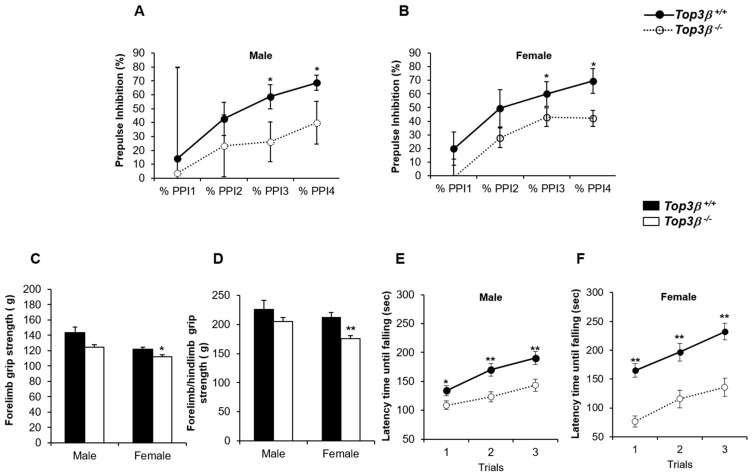
*Top3β^−/−^* mice display neurological defects, lower muscle strength, and altered neuromuscular function. Prepulse-inhibition test was performed with 10 week old mice. Both male (**A**) and female (**B**) *Top3β^−/−^* mice showed reduced percentage of PPI. Grip strengths for forelimb (**C**) and combined forelimb and hindlimb (**D**) were measured for *Top3β^−/−^* and Top3β^+/+^ mice at 9 weeks old. Female *Top3β^−/−^* mice showed reductions for both grip strengths, while male *Top3β^−/−^* mice did not show reduced forelimb grip strength. Six mice per group were used. A rotarod test was carried out with 10 week old *Top3β^−/−^* male (**E**) and female (**F**) mice. The latency until falling off from the start on the accelerating rod was measured with three trials (interval of 15 min). Both sexes of *Top3β^−/−^* mice showed decreased latency. *n* = 6 for each group. * *p* < 0.05; ** *p* < 0.01.

To determine the effect of disruption of the Top3β gene on sensorimotor gating, we measured acoustic pre-pulse inhibition (PPI) in *Top3β^−/−^* mice. PPI is a standard test to determine sensory motor gating function. It is used frequently to evaluate schizophrenia across species, including humans and rodents [23,24]. The PPI was significantly reduced in *Top3β^−/−^* mice compared to WT mice (Figure 5A,B). Since reduction in PPI can serve as a biomarker of schizophrenia [25], neurological deficits of Top3β KO mice suggest that our newly produced *Top3β^−/−^* mouse model might recapitulate symptoms of schizophrenia patients.

We further characterized neuromuscular functions in *Top3β^−/−^* mice by performing grip strength and rotarod tests. Grip strength measurement is frequently used to detect neuromuscular disorders in mouse models [26,27]. The grip strength test was designed to analyze the maximum muscle strength by measuring grip strengths of two forelimbs and combined forelimbs and hind limbs [28]. Only female *Top3β^−/−^* mice showed significantly weaker forelimb grip strengths than their WT littermate mice (Figure 5C). Female, but not male, *Top3β^−/−^* mice also showed significantly weaker combined forelimb and hind limb strength than WT (Figure 5D).

To further determine neuromuscular functions related to motor coordination, balance, motor learning, and cerebellar function, the rotarod test [29,30] was performed with 10 week old *Top3β^−/−^* mice. The latency time until falling from the test rod was measured. *Top3β^−/−^* mice showed a significantly shorter latency time than WT mice (Figure 5E,F), indicating some roles of Top3β in neuromuscular activity and motor coordination of mice. The latency time was increased with more trials conducted using both WT and mutant mice, suggesting their normal motor learning ability. These data indicate that the lack of Top3β is responsible for decreased neuromuscular activities in *Top3β^−/−^* mice.

### 2.8. Central Nervous Tissues Display Top3β Gene Expression

**Figure 6:**
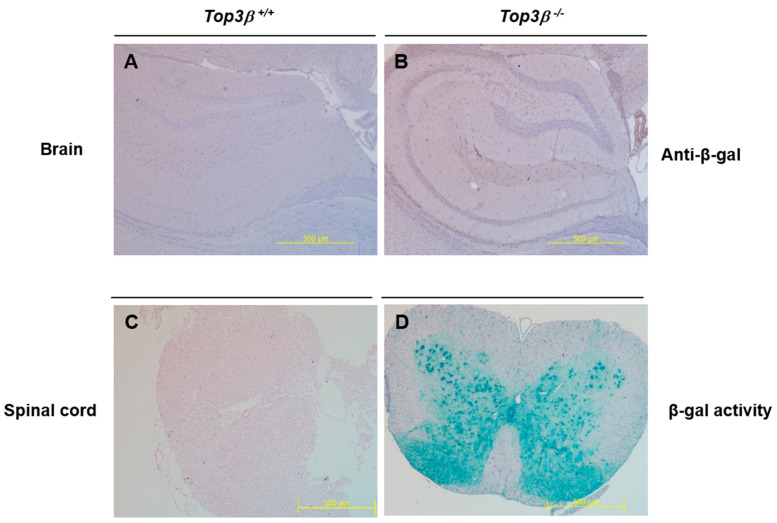
*Top3β* gene expression is clear in the central nervous system. Brain tissue block was cut into 4 μm thicknesses and subjected to immunohistochemistry staining. Meyer’s hematoxylin was used as a counter stain. (**A**,**B**) Immunohistochemistry staining with antibody against β-galactosidase shows abroad positive signal in the brain of *Top3β^−/−^* mouse (×40). (**C**,**D**) X-gal staining in the full spinal cord of *Top3β^−/−^* mouse shows clear β-galactosidase activity in *Top3β^−/−^* mouse (×40).

To determine if the altered behavioral phenotype observed in *Top3β^−/−^* mice was associated with Top3β gene expression, expression of Top3β in the brain and spinal cord was determined by immunohistochemistry. Beta-galactosidase is expressed under the control of endogenous Top3β gene promoter in our *Top3β^−/−^* mice. Therefore, immunohistochemistry was performed for beta-galactosidase and/or X-gal using central nervous tissues obtained from 15 week old *Top3β^−/−^* mice. A broad β-galactosidase expression in the brain of *Top3β^−/−^* mice was confirmed by immunohistochemistry using antibody against β-galactosidase (Figure 6A,B). β-galactosidase expression was also clearly observed in the gray matter of spinal cords of *Top3β^−/−^* mice by X-gal staining (Figure 6C,D). In addition, RT-PCR and Western blot analyses showed a clear Top3β expression in central nervous tissues of WT mice (Figure 1C,D), implying the association of Top3β with neuro-behavioral alterations of *Top3β^−/−^* mice.

### 2.9. Deficiency of Top3β Provokes Altered Connectivity in Brains of Top3β^−/−^ Mice

**Figure 7:**
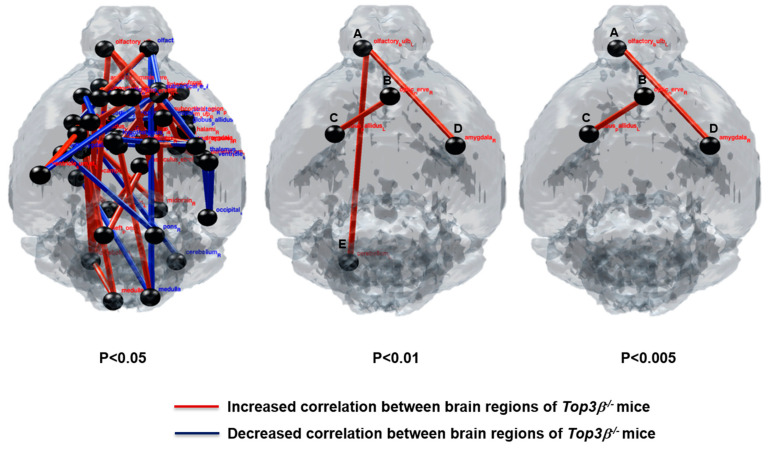
Functional correlation map in different regions of *Top3β^−/−^* mice brains by PET/CT image analysis. Metabolic correlation in brains of *Top3β^−/−^* mice was determined from (^18^F-FDG) PET analysis. *Top3β^−/−^* mice showed altered metabolic correlation in different nodes of brain regions. Red lines and blue lines indicate increased and decreased correlations between two brain regions of 53 VOIs, respectively (*p* < 0.005, *p* < 0.01, and *p* < 0.05 vs. WT mice, *n* = 7 mice per group). A; olfactory bulb, B; optic nerve, C; globus pallidus, D; amygdala, E; cerebellum. L; left, R; right.

To further investigate whether there were any physiological changes in the brain region connections of *Top3β^−/−^* mice, we analyzed functional connectivity between different brain regions using the [^18^F] fluorodeoxyglucose (^18^F-FDG) PET/CT technique. Using PET data, we constructed metabolic and neuronal networks to determine altered correlation between various regions in the brain. The interregional correlation matrices used 53 anatomical volumes of interest (VOIs) in the mouse brain to examine the correlation between two nodes in the brain. A more detailed procedure is available in the Section 4. Surprisingly, *Top3β^−/−^* mice showed altered (increased or decreased) connectivities between different nodes in the brain region as compared to WT mice (Figure 7, Table 1). These differences of connectivity reveled that *Top3β^−/−^* mice had altered correlations indicated by red and blue lines between pairwise VOIs as shown in Figure 7. Alterations of connectivity between the olfactory bulb and the amygdala and connectivity between the globus pallidus and the optic nerve in *Top3β^−/−^* mice brains were found to be more significant (*p* < 0.005) compared to WT. The dysconnectivity and dysfunction of these brain regions were previously reported to be associated with several neurodevelopmental, psychiatric, and behavioral disorders [31,32,33,34,35,36].

By comparing the functional map in different regions of mice brain correlation matrices from (^18^F-FDG) PET analysis (Figure 7), we determined altered metabolic correlation between two nodes in the brain (*p* < 0.05, *p* < 0.01, *p* < 0.005). L; Left, R; Right.

## 3. Discussion

TOP3β is the newest member of the DNA topoisomerase family in mammals [5,6] characterized by their transient cleaving of a DNA strand by transesterification between the 5′-phosphoryl group of a DNA and a tyrosyl group of an enzyme [37,38]. It also acts as an RNA topoisomerase. It is associated with fragile X syndrome known to regulate neuronal functions. Abnormalities in TOP3β are linked to schizophrenia [7], autism [9], mental dysfunction, and intellectual disabilities in humans [12]. Tarsitano et al. [11] previously reported that patients with duplicated TOP3β-containing regions exhibit symptoms such as intellectual disability and show developmental delay and microcephaly. They suggested that this duplicated gene in chromosomes 22q might act as a genomic modifier of its clinical phenotype. In a previous report [16], although *Top3β^−/−^* mice developed and matured without apparent defect, they had shorter lifespans than their *Top3β^+^*^/+^ littermates. Top3β deficiency also increases infertility and aneuploidy in mice, suggesting their role in reproduction [38].

In the present study, we focused on roles and mechanisms of Top3β in regulating mouse behavioral phenotypic makeup. We generated *Top3β^−/−^* mice using the gene trapping method and explored functions of Top3β in a mouse behavioral phenotype. Unlike previously reported *Top3β^−/−^* mice with shorter life spans [16], our mice exhibited normal life spans without showing differences in body weights from their WT littermates (Supplementary Figure S2). Our mice could be a better mutant model to further elaborate undiscovered functions of Top3β at the aged stage of these mice.

Whether other topoisomerases play a compensatory role in the survival and longevity of *Top3β^−/−^* mice is an important question. Thus, we investigated expression of six other topoisomerases enzymes (Top1, Top1mt, Top2α, Top2β, Top3α, and Spo11) [3,4] in *Top3β^+/+^* and *Top3β^−/−^* mice by qRT-PCR. Interestingly, some of these topoisomerases such as Top1, Top1mt, Top2α, Top2β, and Top3α showed significantly higher expression in *Top3β^−/−^* mice than in WT mice (Supplementary Figure S3). Important roles of several topoisomerases in heterochromatin formation that can sufficiently prolong lifespan were reported earlier [39]. These findings suggest that topoisomerases showing increased expression in *Top3β^−/−^* mice might have a compensatory function in the survival and longevity of these mice.

Growing evidence has suggested that Top3β is involved in many brain-related diseases such as schizophrenia, cognitive impairment, autistic spectrum disorders, neurodevelopment and mental dysfunction, juvenile myoclonic epilepsy, and facial dysmorphism in humans [8,9,10,12,13,14]. To ensure that these observations were not specific to just human diseases, we particularly expanded the panel of disorders in the *Top3β^−/−^* mouse model. We assessed the behavioral phenotypes of *Top3β^−/−^* mice with a panel of behavior tests. Results showed that Top3β deficiency significantly affected mouse behavior. As demonstrated by open field and light-dark box tests, *Top3β^−/−^* mice showed lower anxiety levels than their WT littermates (Figure 2). We also found sex differences in the anxiety level and depression level from the open field test and force swim test, respectively. That is, unlike the *Top3β^−/−^* male mice, the *Top3β^−/−^* female mice showed no difference in these tests. According to the extensive review on the sex differences in animal models of psychiatric disorders, in general, female mice exhibit less anxiety than male mice [40]. It is also known that women are more susceptible than men to develop neuropsychiatric disorders including major depression [40]. In this study, the fact that only male mutant mice showed more sensitive phenotype is not concordant with the fact that male animals are more vulnerable than female [40], although we do not understand the reasons for the sex differences observed in our study.

Evidence indicating that disruption of Top3β augments schizophrenia [7,8] and autism [9] in humans suggests that Top3β may contribute to pathogenesis of these mental disorders. PPI deficits are found in various psychiatric populations, most specifically in patients with schizophrenia [41,42]. Similarly, our PPI data showed reduced PPI in *Top3β^−/−^* mice, suggesting that Top3β deficiency could lead to schizophrenia-like behavior in mice. Neuromuscular functions were also affected by Top3β deficiency in mice (Figure 5C–F). Moreover, our forced swim test data revealed that lack of Top3β encouraged anti-depressive-like behavior in these mice besides schizophrenia-like behavior (Figure 3).

We further characterized cognitive functions of *Top3β^−/−^* mice by performing fear conditioning and spontaneous Y-maze tests. However, in both tests, significant differences between mutant and WT mice were not observed, indicating normal learning and memory ability of *Top3β^−/−^* mice (Supplementary Figure S4).

Many previous studies have reported a substantial connection between depression and circadian activity. Circadian factors can beef up the etiology of depression in human and animals [21,22,43,44]. It has been demonstrated that circadian rhythm disruption is common in people with anxiety disorders [45,46,47,48] and that poor sleep or the lack of sleep can also negatively affect a person’s ability to properly function [49]. Here, we also observed altered circadian activity in *Top3β^−/−^* mice. Our results revealed that *Top3β^−/−^* mice were more active during the light time than WT as indicated by increased daytime water intake and less active during the dark time as indicated by reduced dark time locomotive activity (Figure 4). These data further strengthen the idea that there is a strong correlation between depression and altered circadian activity in *Top3β^−/−^* mice.

Recently, it was demonstrated that *Top3β^−/−^* mice show transcriptional and behavioral impairments related to psychiatric disorders and cognitive impairment, suggesting essential roles of Top3β in brain functions [18]. Considering the indispensable role of Top3β in the regulation of neural function [4,7,12,50], we tried to find any brain dysfunction that could be associated with observed behavioral alterations in *Top3β^−/−^* mice. A growing number of studies have also revealed that defects in brain connectivity and functional networks along with disruption in various genes are hallmarks of severe neuropsychiatric-disorders such as autism spectrum disorders, and schizophrenia [51,52,53,54]. We here hypothesize that Top3β is required for normal brain connectivity and that its defect contributes to a behavioral phenotype similar to generalized neurological and mental disorders. A previous study showed that impaired correlation between different regions in the brain is associated with altered neuronal activity and brain dysfunction in rodents [55]. We performed (^18^F-FDG) PET imaging to examine metabolic activities of mice brains. FDG PET images revealed that *Top3β^−/−^* mice displayed altered connectivities in several brain regions. Expression of the *Top3β* gene in the brain implies that the function of Top3β is directly associated with behavioral changes of mice observed in this study. More importantly, altered brain connectivities in *Top3β^−/−^* mice showed homologies with those observed in schizophrenia [56] and the autism mouse model [57,58]. Dysfunction of specific brain regions such as the olfactory bulb is commonly and consistently observed in many neurodegenerative diseases [31,32]. Functional dysconnectivity of olfactory regions in schizophrenia patients serves as a sensitive indicator of schizophrenia pathology [33,59]. Moreover, abnormal structure and function of amygdala have been found to contribute to neurodevelopmental and psychiatric disorders such as autism, anxiety, childhood bipolar, intellectual disability, and schizophrenia [34,35]. Globus pallidus dysfunction can also induce behavioral disorders in primates, indicating its roles in controlling behavioral phenotypes [36]. Consistent with these findings, our study demonstrated a critical role of Top3β in controlling behavioral phenotypes of mice by regulating connectivities of different brain regions. These results clearly indicate that disrupted brain connectivities are correlated with observed neuro-behavioral abnormalities in *Top3β^−/−^ mice.*

## 4. Materials and Methods

### 4.1. Generation of Top3β Mutant Mice

KTPU8 ES cells and pU-21T gene trap vector were used to introduce a mutation in the *Top3β* gene as described previously [60,61]. An ES cell clone with Top3β mutation was chosen by neomycin selection and used to produce a Top3β mutant mouse. The vector insertion site on the genome was determined by nucleotide sequencing as describe previously [60]. The PCR primers used in this study were as follows: common forward primer, 5′-CCCAGCACTCAGGAGTTGAA-3′; mutant specific reverse primer, 5′-TAGCACTTCAGCACAAGGGG-3′; and wild specific reverse primer, 5′-TCCATGCTGAGGATGAGGGA-3′. PCR product sizes for wild and mutant alleles were 550 and 404 base pairs, respectively. Mutant ES cells were introduced into blastocysts from C57BL/6J mice by microinjection. These blastocysts were transferred into pseudo-pregnant mice to get chimeric mice. Obtained chimeric male mice were mated with albino C57BL/6J females to establish germ line transmission of mutant allele. After backcrossing with C57BL/6J at least for six generations, the mice were used for experiments in this study. All animal experiments were conducted with approval from the Institutional Animal Care and Use Committees (IACUC) of the Korea Research Institute of Bioscience and Biotechnology (KRIBB-AEC-1918). They were carried out in accordance with institutional guidelines and regulations (https://www.alio.go.kr/popSusiView21110.do?seq=2016102601282472 (accessed on (17 November 2021)). All animals were bred and maintained with free access to food and water in a specific-pathogen-free facility under a 12 h–12 h light-dark cycle (lights on at 7:00; lights off at 19:00) with temperature of 22 ± 0.5° and humidity of 55 ± 15%.

### 4.2. Real-Time Quantitative Polymerase Chain Reaction (q-PCR)

Fresh isolated tissues from WT and *Top3β^−/−^* mice were treated with Trizol reagent, mixed with 200 µL chloroform, and centrifuged at 4 °C for 15 min at 8000× *g*. The supernatant was recovered, mixed with 500 µL 2-propanol, and centrifuged at 4 °C for 10 min at 8000× *g*. The mRNA pellet was extracted with 75% ethanol to increase RNA purity. After centrifugation, ethanol was removed by evaporation and the pellet was dissolved in diethylpyrocarbonate-treated distilled water (Tech & Innovation, Chuncheon, South Korea). A reverse-transcription aid kit (Thermo Scientific, Foster City, CA, USA) was used to synthesize cDNA, which was then subjected to q-PCR using SYBR Green PCR Master Mix (Roche, Basel, Switzerland) and a LightCycler 480 Instrument II (Roche). GAPDH was used as an internal control. Sequences and sources of all primers used for PCR are listed in Supplementary Table S1 [16,62,63,64,65]. The Spo11 TaqMan probe was used.

### 4.3. Western Blot Analysis

Rabbit anti-Top3β monoclonal antibody (ab183520, Abcam, Cambridge, MA, USA) was purchased from Abcam. Anti-GAPDH antibody was obtained from Cell Signaling (Danvers, MA, USA). Tissue extract (10 µg) was subjected to 10% sodium dodecyl-sulfate polyacrylamide gel electrophoresis (SDS-PAGE) followed by protein transfer to polyvinylidene difluoride (PVDF) membranes (Bio-Rad, Hercules, CA, USA). Membranes were then blocked at room temperature for 1 h with 5% skimmed milk in TBST (50 mM Tris·HCl, pH 7.4, 150 mM NaCl, and 0.1% Tween-20). Afterwards, they were incubated with primary anti-Top3β (dilution of 1:1000) and anti-GAPDH antibodies (dilution of 1:1000) diluted with 1% skimmed milk in TBST. After overnight incubation at 4 °C, secondary antibody (horseradish peroxidase-conjugated anti-rabbit IgG, Cell Signaling, Danvers, MA, USA, 1:1000 dilution) was added to the membranes and incubated at room temperature for 1 h. For signal detection, ECL Super Signal West Dura reagent (Bio-Rad, Hercules, CA, USA) was used.

### 4.4. Open Field Test

The open field test is commonly used to assess exploratory [66] and anxiety-like behaviors [67]. The test used in this study was performed with an open arena made of acrylic box (40 × 40 × 32 cm) with a charge-coupled device (CCD) camera tracking system (O’Hara & Co., Tokyo, Japan). A mouse was placed in a corner of the box. Its activity was recorded for 20 min. Its behavior was then analyzed using a TimeOFCR4 software (O’Hara & Co., Tokyo, Japan).

### 4.5. Light-Dark Box Test

The light-dark box test is another test that can evaluate anxiety-like behavior [68]. The box used in this study was divided into two compartments, a light compartment (27 × 27 × 27 cm) and a dark compartment (27 × 18 × 27 cm), separated by a wall with a small opening (5 × 5 cm). After placing a mouse in the dark compartment, mouse movement in the box was observed for 10 min. The latency time until the first exit from the dark compartment, the time spent in the light compartment, and the number of transitions between the two compartments were measured.

### 4.6. Rotarod Test

We performed a rotarod test using a rotarod 7650 (Ugo Basile Biological Research Apparatus, Barese, Italy) [69] to measure forelimb and hind limb motor coordination, balance, and motor learning ability of mice. Mice were placed on the rotarod at an accelerating speed from 4 rpm to 40 rpm for 300 s. Latency to fall from the rotarod was measured for each animal. The test consisted of three trials with 15 min intervals between trials.

### 4.7. Forced Swim Test

In this study, the forced swim test was based on the Porsolt forced swim test [70,71]. Mice were placed into an acrylic cylinder filled with water at a height of 20 cm for 10 min. The temperature of the water was adjusted to 24 ± 1 °C to avoid hypothermia. The total time that mice spent remaining immobile was measured during the last 4 min of the trial. Immobility was defined as the condition in which a mouse was in a stationary posture with only movements necessary to keep its head above the water.

### 4.8. Y-Maze, Fear Conditioning, and Acoustic Startle and Pre-Pulse Inhibition Tests

Three different behavioral tests were performed using wild and *Top3β^−/−^* mice. Detailed procedures are available at the International Mouse Phenotyping Consortium (IMPC; www.mousephenotype.org (accessed on 17 November 2021)). Some procedures were modified to fit KRIBB laboratory conditions. All phenotyping analyses were conducted at approximately the same time of day. All behavioral tests were performed with the same mice.

### 4.9. X-Gal (5-Bromo-4-chloro-3-indolyl-b–galactopyranoside) Staining

Fresh mouse tissues were fixed with 1% formaldehyde, 0.2% glutaraldehyde, and 0.02% NP-40 in phosphate buffered saline without calcium or magnesium (PBSO) for 2 h at 4 °C and washed with 2 mM MgCl_2_, 0.02% NP-40, and 0.01% Na-Deoxycholate in PBSO for 20 min twice. For the staining to analyze LacZ activity, tissues were shaken overnight in X-gal staining solution (5 mM K_3_Fe(CN)_6_, 5 mM K_4_Fe(CN)_6_·3H_2_O, 2 mM MgCl_2_, 0.02% NP40, 0.1% sodium deoxycholate, and 1 mg/mL X-gal in PBS). After removing the solution, tissues were washed with PBS for 20 min twice and fixed with 4% paraformaldehyde in PBSO at 4 °C overnight. After washing with PBS for 20 min twice, these tissues were dehydrated by serial washing with 70% ethanol for 1 h twice, 97% ethanol for 1 h twice, and 100% ethanol for 1 h twice. After rinsing with N-butanol for 1 h, these tissues were embedded with paraffin. Subsequently, tissue sections were prepared 4 μm in thickness. After deparaffinization and rehydration, slides were stained with nuclear fast red and dehydrated with serial ethanol and xylene. The slides were finally mounted and covered with coverslips.

### 4.10. Immunohistochemical Staining against β-Galactosidase

Mouse tissues were fixed with 10% neutral formaldehyde solution for at least 48 h and then embedded with paraffin. Tissue blocks were sectioned (4 μm in thickness). Tissue sections were deparaffined, rehydrated as described above, and then treated with 1X antigen retrieval solution (pH 6.0, DAKO., Hayward, CA, USA) through high temperature–pressure by autoclaving for 30 min. The section slides were kept in the solution until they became transparent at room temperature. They were then rinsed with distilled water (DW) and PBSO for 5 min each. Slides were then treated with antigen blocking solution (DAKO., Hayward, CA, USA) for 2 h and then incubated with primary antibody against β-galactosidase (1:700, Hayward, CA, USA) at 4 °C overnight. After washing with 1X PBSO three times for 5 min each, slides were treated with secondary antibody (DAKO., Hayward, CA, USA) for 15 min. Positive signals were visualized with a chromogen (DAKO., Hayward, CA, USA) according to the manufacturer’s guide. A counter staining was performed for these slides with hematoxylin.

### 4.11. PET Image Acquisition

Mice were fasted for at least 6 h prior to PET image acquisition. A heating pad was placed on the bed to keep it warm during the PET scan. The head was fixed with a band to prevent movement.

Dynamic PET images were obtained simultaneously with an [^18^F]-fluorodeoxyglucose (^18^F-FDG) bolus injection (12.21 ± 0.42 MBq in 0.2 mL saline) into a lateral tail vein for 90 min. During PET image acquisition, mice were anesthetized with isoflurane (2.5% flow rate).

For each PET scan frame, 44 transaxial images (43 × 32 pixel; 0.6 mm pixel size; 0.6 mm plane thickness) were reconstructed with an ordered subset using an expectation maximization iterative algorithm (4 iterations, 3 subsets). Average data at 40–90 min after injection were used for the analysis.

### 4.12. Image Processing

Cropping and a template-based rigid co-registration into mouse brain PET template (created by the laboratory itself, 0.2 mm × 0.2 mm × 0.2 mm) space were performed using a PMOD medical image analysis software (PMOD Technologies LLC, RRID: SCR_016547, v4.1, Zürich, Swiss). An isotropic Gaussian filter with 0.4 mm full width half maximum (FWHM) and reference count normalization were applied to obtain a standard uptake value ratio (SUVR) map to achieve comparability between all images. In this study, medulla was used as the reference region.

Volume-of-interest (VOI) was compared for 53 areas (including left and right). Significant differences in (^18^F-FDG) uptake (as SUVR) between *Top3β^−/−^* and WT groups were detected using SnPM toolbox, a non-parametric permutation test.

To perform brain functional network analysis, we used 53 nodes represented by 53 volumes of interest (VOIs) [72]. We extracted intensity-normalized FDG uptake in VOIs of each mouse. With FDG uptake, correlation coefficients were obtained. Pearson’s correlation coefficients (r) between each pair of VOIs were calculated in an inter-subject manner and a correlation matrix (53 53) was obtained from each group [55].

### 4.13. Statistical Analysis

Statistical analyses for the results obtained from behavior tests were performed by unpaired and two-tailed Mann–Whitney U test using GraphPad Prim (version 7.04, San Diego, CA, USA). Statistical significance was accepted when the *p* value shows <0.05 as significant. Data were represented as means ± standard error of the mean (S.E.M). The statistical analysis for interregional correlations between groups for PET results and permutation tests for all possible connections between nodes were performed. The interregional correlation matrixes of all groups were transformed to Z scores by using Fisher transformation. For all 53 VOIs, randomly reassigned labels were permuted 5000 times. Followed by Fisher transformation interregional correlation matrices were then calculated accordingly. We obtained type I error from comparison between the observed Z score for each connection and the Z score from permuted data. At *p* < 0.05, the threshold was set to determine statistically different connections between groups.

## 5. Conclusions

Abnormalities of topoisomerase IIIβ (Top3β) are linked to schizophrenia, autism, mental dysfunction, and intellectual disabilities in humans. However, there is no report showing clear associations of Top3β with neuropsychiatric phenotypes in mice. Here, we report that *Top3β^−/−^* mice show decreased anxiety and depression-like behaviors and that the lack of Top3β is associated with changes in circadian rhythm. We also confirmed significant altered connectivities between many brain regions in *Top3β^−/−^* mice, including the connectivity between the olfactory bulb and the cerebellum, the connectivity between the amygdala and the olfactory bulb, and the connectivity between the globus pallidus and the optic nerve. These results suggest that Top3β is associated with neuropsychiatric disorders such as schizophrenia, autism, and mental dysfunction in mice as in humans.

In conclusion, we demonstrate that Top3β deficiency is associated with brain network disorders in mice and that Top3β could be an interesting target to study neuropsychiatric disorders in humans.

## Figures and Tables

**Table 1:** Altered connectivity between two brain nodes in *Top3β^−/−^* mice compared with that in wild mice based on PET/CT analysis.

Increased Correlation between Two Nodes		Decreased Correlation between Two Nodes		*p*-Value
optic_nerve_R	frontal_lobe_R	optic_nerve_R	optic_nerve_L	*p* < 0.05
optic_nerve_R	midbrain_R	olfactory_bulb_R	anterior_commissure_R	
optic_nerve_R	globus_pallidus_L	olfactory_bulb_R	medulla_R	
olfactory_bulb_R	subcortical_region_frontal_L	olfactory_bulb_R	striatum_L	
anterior_commissure_R	midbrain_R	anterior_commissure_R	ventricle_sum_lat_L	
anterior_commissure_R	mamilothalamic_tract_L	occipital_cortex_R	thalamus_R	
anterior_commissure_R	corpus_callosum_L	occipital_cortex_R	ventricle_sum_lat_R	
frontal_lobe_R	stria_medullaris_thalami_L	subcortical_region_frontal_R	globus_pallidus_R	
parietal_temporal_cortex_R	stria_medullaris_thalami_R	subcortical_region_frontal_R	amygdala_R	
subcortical_region_parieto_temopal_R	internal_capsule_R	subcortical_region_frontal_R	corpus_callosum_L	
fasciculus_retroflexus_R	cerebellum_L	striatum_R	ventricle_sum_sup_L	
fasciculus_retroflexus_R	left_pons_L	internal_capsule_R	hypothalamus_L	
fasciculus_retroflexus_R	midbrain_L	mammilothalamic_R	corpus_callosum_L	
fornix_R	internal_capsule_R	amygdala_R	corpus_callosum_L	
globus_pallidus_R	internal_capsule_R	medulla_R	hippocampus_L	
stria_medullaris_thalami_R	amygdala_R	pons_R	left_amygdala_L	
midbrain_R	corpus_callosum_L	cerebellum_R	corpus_callosum_L	
amygdala_R	olfactory_bulb_L	ventricle_sum_sup_R	corpus_callosum_L	
medulla_R	mamilothalamic_tract_L	subcortical_region_parieto_temopal_L	frontal_lobe_L	
ventricle_sum_sup_R	olfactory_bulb_L			
ventricle_sum_lat_L	globus_pallidus_L			
cerebellum_L	medulla_L			
cerebellum_L	internal_capsule_L			
cerebellum_L	subcortical_region_parieto_temopal_L			
cerebellum_L	frontal_lobe_L			
cerebellum_L	olfactory_bulb_L			
medulla_L	internal_capsule_L			
hippocampus_L	midbrain_L			
globus_pallidus_L	striatum_L			
subcortical_region_frontal_L	anterior_commissure_L			
optic_nerve_R	globus_pallidus_L			
amygdala_R	olfactory_bulb_L			
Cerebellum_L	olfactory_bulb_L			*p* < 0.01
Optic_nerve_R	globus_pallidus_L			*p* < 0.005
amygdala_R	olfactory_bulb_L			

## Data Availability

Data is contained within the article.

## Supplementary Materials

The following are available online at https://www.mdpi.com/article/10.3390/ijms222312806/s1.
