# Supplementary material for: Topoisomerase IIIβ Deficiency Induces Neuro-Behavioral Changes and Brain Connectivity Alterations in Mice
Source: Int J Mol Sci. 2021 Nov 26;22(23):12806. doi: 10.3390/ijms222312806 (PMC8657541; doi:10.3390/ijms222312806)
Supplement: Supplementary file 1 [file ijms-22-12806-s001.zip › ijms-1440113-supplementary.pdf]

Supplementary Materials

Topoisomerase IIIβ Deficiency Induces Neuro-Behavioral Changes and Brain Connectivity Alterations in Mice

Faiz Ur Rahman,[1] You-Rim Kim,[1,2] Eun-Kyeung Kim,[1] Hae-rim Kim,[1] Sang-Mi Cho,[1] Chin-Soo Lee,[1] Su Jin Kim,[4] Kimi Araki,[3] Ken-ichi Yamamura,[3] Mini Lee,[1] Seul Gi Park,[1] Won-Kee Yoon,[1] Kihoon Lee,[1] Young-Suk Won,[1] Hyoung-Chin Kim,[1] Younghee Lee,[2] Ho-Young Lee,[4] and Ki-Hoan Nam[1]

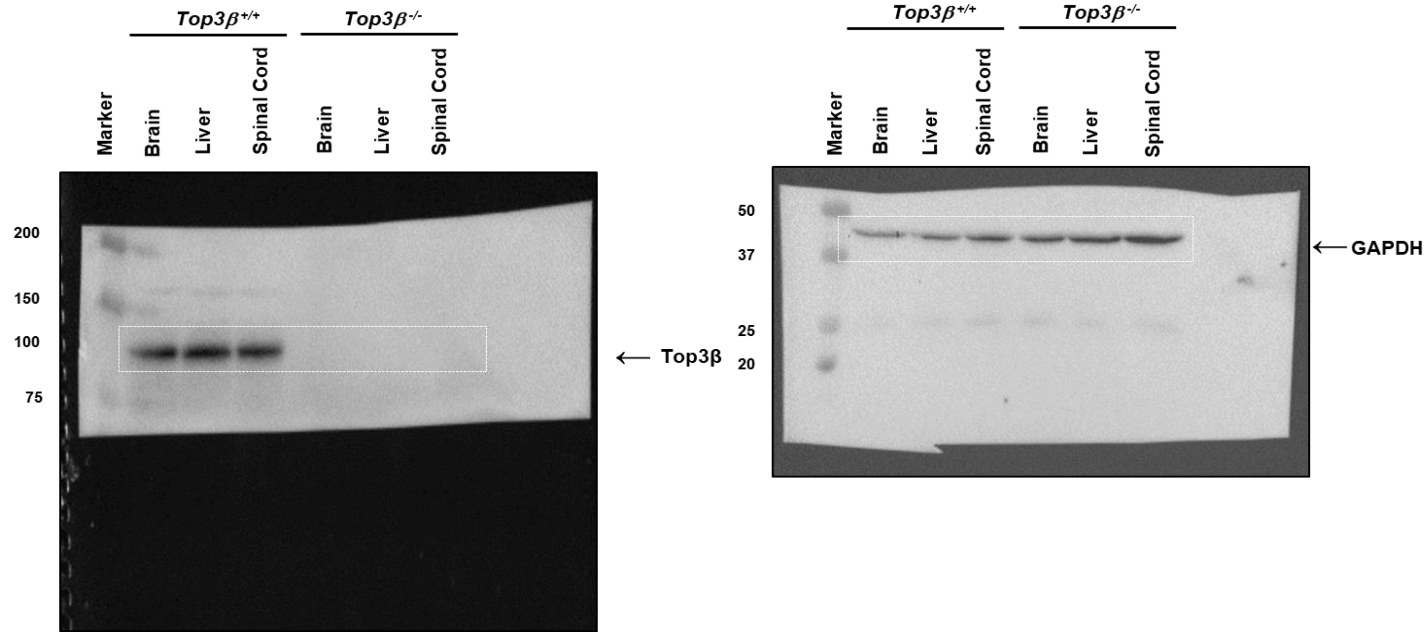

Figure S1. Full blot images of Top3β and GAPDH expression. Full blot images of Top3β (upper panel) and GAPDH (lower panel) expression in brain, liver, and spinal cord lysates of *Top3β*$^{+/+}$ and *Top3β*$^{-/-}$ mice are shown.

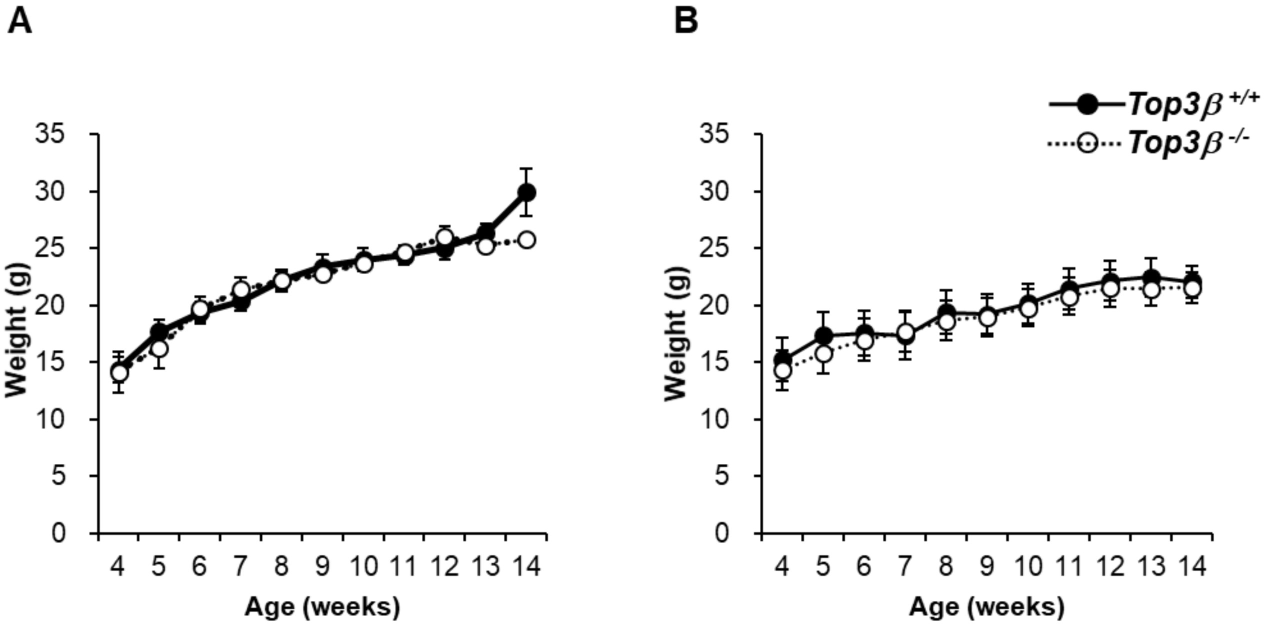

Figure S2. Body weight gain curve of *Top3β*$^{-/-}$ mice. Body weight gains were measured every week from 4 to 16 weeks of age for both sexes of *Top3β*$^{-/-}$ and wild type mice. A, male; B, female. N = 6 for each male group and N = 5 for each female group.

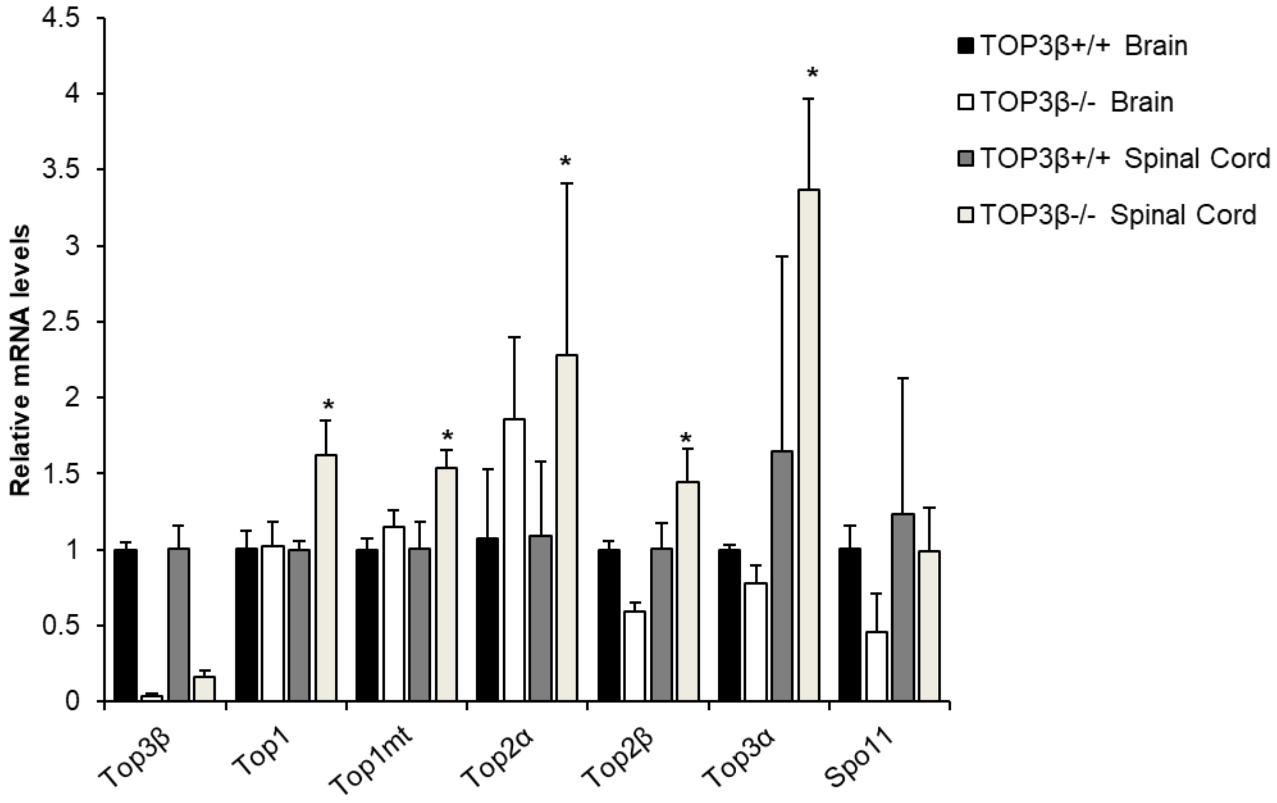

Figure S3. Comparative expression analysis of different topoisomerase enzymes in *Top3β*$^{+/+}$ and *Top3β*$^{-/-}$ mice. Expression analysis of mRNA encoding Top3β, Top1, Top1mt, Top2α, Top2β, Top3α, and Spo11 in brains and spinal cords of *Top3β*$^{+/+}$ and *Top3β*$^{-/-}$ mice (as measured by qRT-PCR). *, $p < 0.05$.

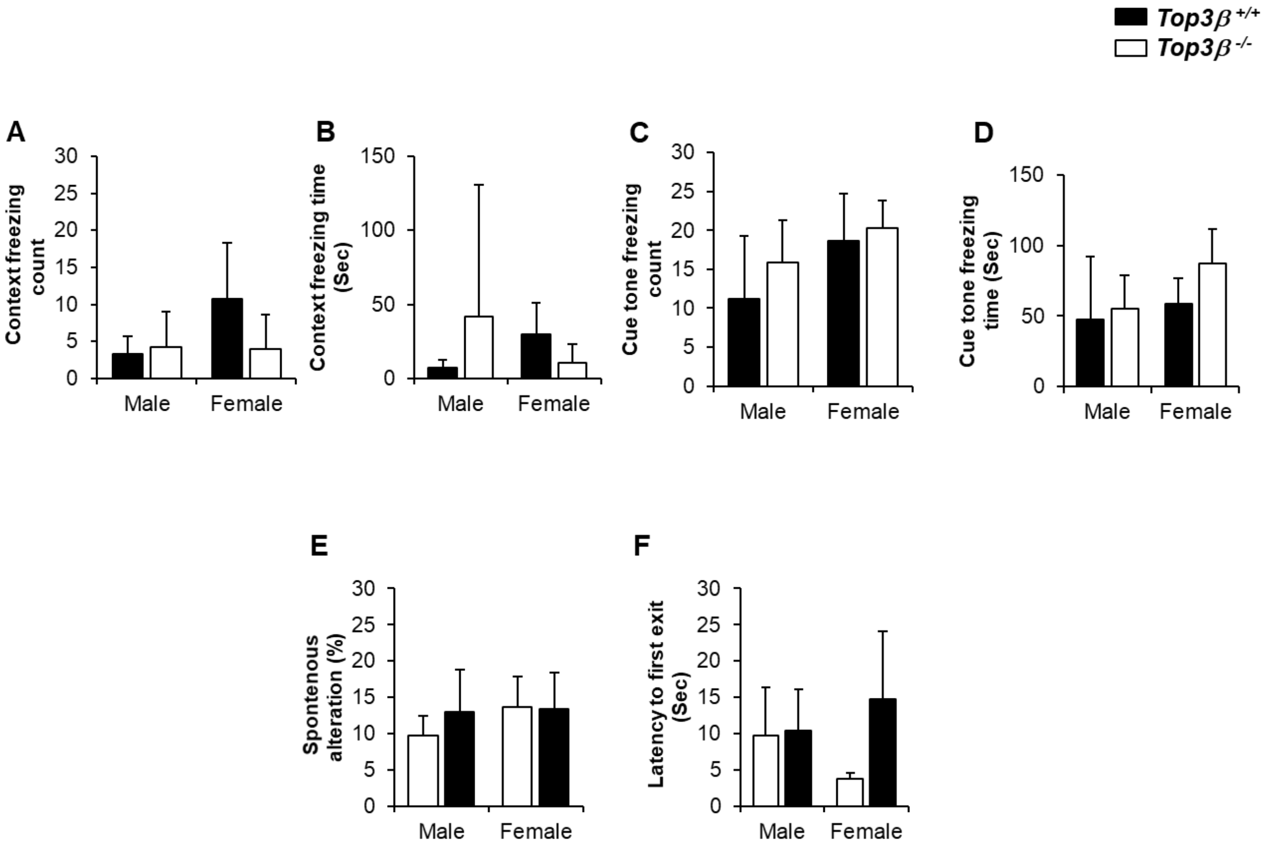

Figure S4. Fear conditioning and Y-Maze are normal in *Top3β*$^{-/-}$ mice. Significant differences in fear conditioning (A-D) and Y-maze tests (E-F) were not observed in 11 weeks old WT or *Top3β*$^{-/-}$ mice. N = 6 for each group.

Table S1. Primers used for real-time PCR in this study.

Species	Target name	Direction	Primer sequence	Source
**Mouse**	**Gapdh**	Forward	CGTGCCGCCTGGAGAAACC	(This study)
		Reverse	TGGAAGAGTGGGAGTTGCTGTTG	
	**Top3β**	Forward	GGAGATTGCACAGATGTTTTTAAAC	(Kelvin Y. Kwan and James C. Wang. et al. 2001)[16]
		Reverse	TTCTGTCCGTGGGTAGCTGATATAGC	
	**Top1**	Forward	GGTGAGAAGGACTGGCAGAAAT	(Liu, L.-M. et al. 2018)[62]
		Reverse	CTTGTCGATGAAGTACAGGGCTA	
	**Top1mt**	Forward	CCTTGACAAATGTGACTTCACG	(Douarre, C. et al. 2012)[63]
		Reverse	GACCACATCCTCTGGCATGAC	
	**Top2α**	Forward	CGGAATGACAAGCGAGAAGTAA	(Liu, L.-M. et al. 2018)[62]
		Reverse	GCATTGTAAAGATGTATCGTGGAC	
	**Top2β**	Forward	TGGTGAGATATTTGTGGTGGACAG	(Tiwari, V. K. et al. 2012)[64]
		Reverse	TGCCGGTGTTTTATCTGTTCCATT	
	**Top3α**	Forward	TCTCTGGACATTTGCTGGC	(Guiraldelli, M. F. et al. 2013)[65]
		Reverse	TGCTTCAAAGAGGACAAGGG	
